# Experimental evidence for heat plume‐induced cavitation and xylem deformation as a mechanism of rapid post‐fire tree mortality

**DOI:** 10.1111/nph.13979

**Published:** 2016-05-06

**Authors:** Adam G. West, Jacques A. Nel, William J. Bond, Jeremy J. Midgley

**Affiliations:** ^1^ Department of Biological SciencesUniversity of Cape TownRondebosch7700South Africa

**Keywords:** cavitation, *Eucalyptus cladocalyx*, fire, hydraulic failure, *Kiggelaria africana*, pyrohydraulics, tree mortality, xylem deformation

## Abstract

- Recent work suggests that hydraulic mechanisms, rather than cambium necrosis, may account for rapid post‐fire tree mortality. We experimentally tested for xylem cavitation, as a result of exposure to high‐vapour‐deficit (*D*) heat plumes, and permanent xylem deformation, as a result of thermal softening of lignin, in two tree species differing in fire tolerance.
- We measured percentage loss of conductance (PLC) in distal branches that had been exposed to high‐*D* heat plumes or immersed in hot water baths (high temperature, but not *D*). Results were compared with predictions from a parameterized hydraulic model. Physical damage to the xylem was examined microscopically.
- Both species suffered *c*. 80% PLC when exposed to a 100°C plume. However, at 70°C, the fire‐sensitive *Kiggelaria africana* suffered lower PLC (49%) than the fire‐resistant *Eucalytpus cladocalyx* (80%). Model simulations suggested that differences in PLC between species were a result of greater hydraulic segmentation in *E. cladocalyx*. *Kiggelaria africana* suffered considerable PLC (59%), as a result of heat‐induced xylem deformation, in the water bath treatments, but *E. cladocalyx* did not.
- We suggest that a suite of ‘pyrohydraulic’ traits, including hydraulic segmentation and heat sensitivity of the xylem, may help to explain why some tree species experience rapid post‐fire mortality after low‐intensity fires and others do not.

## Introduction

The mechanism by which trees die following exposure to a low‐intensity fire is poorly understood. Low‐intensity fires (defined here as fires that do not consume all live aerial biomass) are a regular feature of many ecosystems worldwide. Regular low‐intensity fires may stimulate recruitment (Bond & Keeley, 2005), alter microbial communities (Hart *et al*., 2005), increase nutrient cycling (Grogan *et al*., 2000; Pellegrini *et al*., 2015) and improve resistance to herbivore attack (Hood *et al*., 2015), amongst other factors. However, low‐intensity fires may also result in tree mortality, maintaining open ecosystems, such as savannas, from conversion to closed canopy forest (Bond & Midgley, 2000; Hoffmann & Solbrig, 2003; Hoffmann *et al*., 2009). The ability of a tree to survive exposure to low‐intensity fire is a key determinant of whether these open ecosystems are converted to closed forest (Bond & Midgley, 2000).

The question of why some trees are killed by fire, and others survive, has been the focus of considerable research (Michaletz & Johnson, 2007; Kavanagh *et al*., 2010; Midgley *et al*., 2011; Higgins *et al*., 2012; Michaletz *et al*., 2012), yet the exact mechanisms have remained elusive. It has been largely assumed that fire mortality arises through prolonged lethal heating (> 60°C) of the vascular cambium and phloem of the stem (Dickinson *et al*., 2004; Michaletz & Johnson, 2007). In the event of necrosis of the entire circumference of the vascular cambium and phloem, the tree is unable to transport photosynthate to the roots, ultimately resulting in starvation of these tissues and tree mortality (Bond & Van Wilgen, 1996).

However, there is mounting evidence that hydraulic failure, rather than cambium necrosis, may be a more important mechanism of post‐fire tree mortality (Michaletz & Johnson, 2007; Kavanagh *et al*., 2010; Midgley *et al*., 2011; Michaletz *et al*., 2012). Although there is evidence that trees can delay the cambium from attaining lethal temperatures through insulation of their stem with thick bark (Hoffmann *et al*., 2009; Lawes *et al*., 2011), there are contrary examples that illustrate that bark thickness is not correlated with fire mortality (Midgley *et al*., 2010; Higgins *et al*., 2012; Lawes *et al*., 2014). There is also evidence of heating‐related reductions in xylem conductivity (Ducrey *et al*., 1996; Balfour & Midgley, 2006; Michaletz *et al*., 2012), which may ultimately result in mortality if sufficiently severe. However, perhaps the most compelling evidence of the importance of post‐fire hydraulic failure is that trees often die within days or weeks of a fire (Ducrey *et al*., 1996; Midgley *et al*., 2011), yet trees with full‐circumference ring‐barking (mimicking cambium necrosis) take far longer to die (Midgley *et al*., 2011).

Recently, two distinct, but complementary, mechanisms for post‐fire hydraulic failure have been proposed. These consider increased vulnerability to cavitation and reduced hydraulic conductance as a result of the direct effects of heating on the xylem (Michaletz *et al*., 2012), as well as high levels of cavitation as a result of the indirect effects of being exposed to a heat plume of hot, dry air (Kavanagh *et al*., 2010).

Cavitation in the xylem generally occurs through the mechanism of air seeding, where air is aspirated into a water‐filled conduit, rendering it unable to further transport water (Sperry & Tyree, 1988; Cochard *et al*., 1992). Cavitation occurs when the pressure difference between the air (*P*_air_) and sap (*P*_sap_) is sufficiently large to exceed the pressure required for air seeding (*P*_cav_) (Tyree & Zimmermann, 2002; Michaletz *et al*., 2012):

(Eqn 1) $$P_{\mathrm{air}}-P_{\mathrm{sap}}>P_{\mathrm{cav}}$$

As *P*_air_ does not vary considerably with temperature (Michaletz *et al*., 2012), *P*_cav_ and *P*_sap_ are the critical terms determining cavitation. Heating of the xylem may influence both *P*_sap_ and *P*_cav_.

The cavitation pressure (*P*_cav_) is related to (Tyree & Zimmermann, 2002):

(Eqn 2) $$P_{\mathrm{cav}}=\frac{4\sigma \text{cos}\left(\theta \right)}{D_{\mathrm{pore}}}$$

where σ is the surface tension of water, θ is the contact angle of the meniscus and the wall, and *D*_pore_ is the diameter of the pit pore. As demonstrated by Michaletz *et al*. (2012), heating of the xylem results in a reduction in σ, decreasing *P*_cav_. Heating of the xylem may also increase *D*_pore_ as a result of the thermal softening of lignin, which might allow the cellulose fibres of the pit membrane to shift, reducing *P*_cav_. However, a reduced *P*_cav_ caused by heating alone has yet to be shown experimentally (Michaletz *et al*., 2012).

The other critical term determining cavitation, *P*_sap_, can be approximated as the xylem pressure potential (Ψ_x_). Using derivatives of Darcy's law, Ψ_x_ (MPa) can be shown to be (Jones & Sutherland, 1991; Whitehead, 1998; Kavanagh *et al*., 2010):

(Eqn 3) $$\Psi_{x}=\Psi_{s}-\left(g_{s}cD\right)/K_{\mathrm{leaf}}$$

where Ψ_s_ is the soil water potential (MPa), *g*_s_ is the canopy stomatal conductance (mmol m^−2^ s^−1^ MPa^−1^), *D* is the vapour pressure deficit (kPa), *c* is 1/*P*_air_ (kPa^−1^) and *K*_leaf_ is the leaf specific canopy conductance (mmol m^−2^ s^−1^ MPa^−1^).

Exposure of a canopy to a plume of high *D*, under constant *g*_s_ and *K*_leaf_, would make Ψ_x_ more negative, possibly to the point at which Ψ_x_ exceeds *P*_cav_, resulting in cavitation. Fire plumes can attain high vapour pressure deficits (> 270 kPa) that far exceed the normal atmospheric conditions (< 6 kPa) to which a plant would be exposed (Kavanagh *et al*., 2010). Under natural atmospheric conditions, plants respond to increasing vapour pressure deficit by stomatal closure (Oren *et al*., 2001), thereby preventing excessively negative Ψ_x_ and thus cavitation. Stomatal closure half‐times for angiosperm trees are on the order of 9–29 min (Brodribb *et al*., 2009). In the case of a fast‐moving fire plume, the stomata may not have the time to close before being exposed to the hot and dry plume. Without compensatory stomatal closure, the increasing transpiration rates caused by high vapour pressure deficit would theoretically result in Ψ_x_ falling sufficiently to cause cavitation of the xylem (Kavanagh *et al*., 2010). However, plume‐induced cavitation has yet to be demonstrated experimentally.

In addition to the effects of heating on *P*_sap_ and *P*_cav_ described earlier, the direct effects of heating on the xylem can result in permanent deformation of the conduits, presumably through the thermal softening of lignin which causes conduit collapse (Michaletz *et al*., 2012). This deformation is solidified on cooling, rendering the xylem permanently dysfunctional.

Theoretically, the two direct and indirect effects described earlier could combine to result in high levels of cavitation in the xylem, together with potentially permanently deformed xylem that would be unable to refill and regain full function post‐fire. Given sufficient damage to the xylem, this could result in permanent hydraulic failure for the tree and, ultimately, death.

In this study, we experimentally tested for the relative importance of vapour pressure deficit‐induced cavitation and heat‐induced xylem deformation on post‐fire hydraulic conductance. The two species selected for this study represented extremes of fire survival strategies. *Eucalyptus cladocalyx* is able to survive fires, resprouting epicormically post‐burn (Burrows, 2000; Ruthrof *et al*., 2003). *Kiggelaria africana*, an Afromontane forest species, is killed by fire (Van Wilgen *et al*., 1992). If hydraulic failure is an important component of post‐fire mortality, we would predict that these species would show different responses to the heat treatments, with *K. africana* experiencing a greater degree of permanent damage than *E. cladocalyx*. Specifically, we hypothesized that both species would experience cavitation when exposed to a simulated heat plume, but that the cavitation would be reversible in the fire survivor *E. cladocalyx*, but not in the fire‐sensitive *K. africana*.

In order to test this, we exposed stems to simulated heat plumes at two different temperatures and measured the loss of hydraulic conductance. The lower temperature (70°C) provided sufficiently low vapour pressure deficit to induce cavitation, but was not sufficiently hot to induce deformation. The upper temperature (100°C) provided sufficient heat for both mechanisms to occur. If heat‐induced deformation was an important component of loss of conductance, the two temperature treatments should differ, with a greater loss of conductance at the higher temperature. Furthermore, we tested the extent to which this loss of conductance was recoverable on flushing. We reasoned that a full recovery of conductance would indicate that the loss of conductance was caused by cavitation only, whereas incomplete recovery would indicate some degree of permanent heat‐induced damage to the xylem. This was then verified microscopically.

To further explore the role of direct heating on the stem without the effect of vapour pressure deficit, we immersed stems into a water bath of similar temperatures to the simulated heat plume and measured conductance immediately post‐treatment and again after flushing. We reasoned that a post‐treatment loss of conductance could only be caused by deformation of the xylem because of direct heating, and that, if this was the case, it should not be recoverable post‐flushing. Our experimental results were then compared with a biophysical model of loss of conductance with temperature that was parameterized with data collected for the species in question.

Our study provides the first direct experimental evidence for fire plume‐induced cavitation and demonstrates differential heat‐induced deformation in two species. We suggest that a suite of ‘pyrohydraulic’ traits, including hydraulic segmentation (*sensu* Zimmerman, 1983), thermal sensitivity of xylem deformation and stomatal sensitivity to heat plumes, may be important traits that contribute towards fire survival or mortality.

## Materials and Methods

### Field collection

Samples of *Eucalyptus cladocalyx* (F. Muell.) and *Kiggelaria africana* (L.) were collected adjacent to the Department of Biological Sciences, University of Cape Town. Before sampling, stem water potential (Scholander Pressure Chamber; PMS Instruments, Corvallis, OR, USA) and stomatal conductance (AP4 Porometer; Delta‐T, Cambridge, UK) were measured to ensure that the trees were well hydrated and not overly water stressed. The maximum vessel length for both species was determined by perfusing pressurized air (0.1 MPa) into the proximal end of branch segments whilst placing the distal end into a bucket of water. The distal end was then progressively cut back by 1 cm until bubbles could be seen streaming from the cut surface. The maximum vessel length was then assumed to be roughly the same length as the stem segment remaining. For each species, branches of *c*. 2 m in length were sampled from individual trees, as this ensured that the segments were much longer than the maximum vessel length of each species (61 cm for *E. cladocalyx* and 56 cm for *K. africana*). Branches were cut under water, in order to prevent accidental embolism, and then transported to the laboratory with the cut end submerged in distilled water.

Samples were kept in a phytotron chamber mimicking late summer conditions (14 h 25°C : 10 h 15°C, day : night) where they remained for 24 h in order to acclimatize to the same initial conditions. After 24 h had elapsed, water potential and stomatal conductance were re‐measured. Branches that were actively transpiring with minimal water stress were used for further experimentation. For all measurements and treatments, six replicates, taken from different branches, were used.

### Determination of leaf specific conductance (*K*_leaf_)

Whole‐shoot conductance measurements were made following the methods of Kolb *et al*. (1996). Briefly, shoots were defoliated under water by removing the lamina at its junction with the petiole, leaving as much of the petiole intact as possible. This ensured that even the most distal portions of the xylem vessels, which may be most prone to hydraulic failure, were incorporated into the measurement. The base of the shoot was fitted via tubing to a beaker containing a supply of 0.01 M KCl in reverse osmosis (RO) water, which had been filtered to 0.22 μm. The whole shoot was then inserted into a cylindrical vacuum chamber with only the base of the stem with the tubing protruding out, whilst ensuring an airtight seal using a diameter‐adjustable rubber connector. The tubing was connected to a reservoir of KCl on an electronic balance. Once the shoot had been inserted into the chamber, a partial vacuum was pulled on the chamber, pulling water from the balance reservoir through the xylem. The steady‐state flow rate (kg s^−1^) was logged at a variety of pressures (20–60 kPa), and the slope of the flow rate and pressure was used to determine the hydraulic conductance (kg s^−1^ MPa^−1^). Leaves from the defoliated shoot were measured for leaf area by scanning and analysing with Image J (http://rsbweb.nih.gov/ij/). *K*_leaf_ (mmol m^−2^ s^−1^ MPa^−1^) was calculated as the hydraulic conductance divided by the leaf area.

### Treatments

Three treatments were designed in order to test for direct heating effects and vapour pressure deficit effects separately. For all treatments, shoots were acclimated for 24 h in the phytotron before commencing the treatment.

#### Control treatment

No manipulation was imposed. Shoots were defoliated and *K*_leaf_ was determined. The shoots were then flushed with 0.22‐μm filtered, 0.01 M KCl at 175 kPa for 1 h, and *K*_leaf_ was re‐determined.

#### Simulated heat plume treatment

This treatment tested for the combined effects of both *D* and direct heating. Shoots had their cut end wrapped with Parafilm^™^ to prevent water loss, and were inserted into a beaker filled with polystyrene to eliminate direct conductive heating of the stem through contact with the glass beaker. Shoots were placed into a convection oven at either 70 or 100°C for 6 min with all foliage still intact on the shoot. The 6‐min oven treatment was used as surrogate for a hot, dry plume generated above a fast‐moving surface fire during combustion. After the plume treatment, stems were defoliated and *K*_leaf_ was measured exactly as for the controls.

#### Water bath treatment

To measure the effect of direct heating on the stem alone, without any potential transpiration‐induced cavitation, intact shoots were submerged in a water bath at either 70 or 100°C for 6 min. After the water bath treatment, stems were defoliated and *K*_leaf_ was measured as before.

### Sapwood temperature

Sapwood temperature was measured in a subset of branches (not used for *K*_leaf_ determination) using Type‐T thermocouples embedded in the sapwood and connected to a data logger (CR10x; Campbell Scientific, Logan, UT, USA). Large (0.6–0.9 cm in diameter) and small (< 0.6 cm in diameter) shoots were instrumented and then serially exposed to the simulated plume treatment and water bath treatment, with sufficient cooling time between treatments, in order to compare the effect of the heating treatments on sapwood temperature.

### Microscopy

Two methods of microscopy were used to determine whether any physical damage had occurred in the xylem vessels of both species when subjected to heating treatments.

#### Light microscopy

Samples were sectioned into 6‐μm‐thick cross‐sections using a microtome and stained with Toluidine Blue O. Sections were examined at × 40 and × 100 (oil) using a Nikon Eclipse 50i Compound Microscope (Nikon, Tokyo, Japan). A Nikon DS Camera Control Unit DS‐U2 and DS‐5M Camera Head were used to capture images, which were then processed using NIS Elements Documentation software (Nikon).

#### Scanning electron microscopy (SEM)

Fresh samples, sectioned using a microtome, were examined using a Phenom ProX Desktop Scanning Electron Microscope (Eindhoven, the Netherlands) and processed using Elemental Identification (EID) software package.

### Vulnerability curves

Vulnerability curves for both *E. cladocalyx* and *K. africana* were determined by air injection (Sperry & Ikeda, 1997). We collected branch segments longer than the maximum vessel length for each species (Cochard *et al*., 2013). Segments collected were then flushed for 1 h using 0.01 M KCl at 175 kPa in order to remove any emboli. Segments were cut at both ends and inserted into a pressure sleeve attached to a standard Scholander Pressure Chamber (PMS Instruments). Bark was removed around the entire circumference of the stem portion inserted into the pressure sleeve to facilitate the entry of air into the xylem. The pressure sleeve was pressurized to the target pressure for 10 min before allowing a period of relaxation to prevent outgassing from the cut end during water flow measurements. Percentage loss of conductance (PLC) data were fatigue corrected relative to −0.5 MPa (Hacke *et al*., 2000). PLC curves for each species were fitted and tested for significant difference following the procedures of Pammenter & van der Willigen (1998). This approach results in two fitted parameters (*a* and *b*) that define the vulnerability curve, with *b* approximating the pressure at 50% loss of conductance, or *P*_50_ (Pammenter & van der Willigen, 1998).

### Model description

We modelled the effects of a heat plume on the percentage loss of leaf specific canopy conductance (*K*_leaf_) using a hydraulic model (Supporting Information Fig. S1; Table S1). A more complete description of heat plume modelling can be found in Kavanagh *et al*. (2010). For the purposes of this model, the heat plume was modelled as a vector of increasing temperatures, assuming a constant relative humidity of 0% (Kremens *et al*., 2003), resulting in a vector of increasing vapour pressure deficit (*D*, kPa):

(Eqn 4) $$D=e_{s}-e_{a}=\left(0.611\text{exp}\left(\left(17.27T\right)/\left(237.3+T\right)\right)\right)*\left(1-\text{RH}/100\right)$$

where *e*_s_ is the saturation vapour pressure (kPa), *e*_a_ is the actual vapour pressure (kPa), *T* is the temperature (°C) and RH is the relative humidity (%) (Campbell & Norman, 1998).

At each independent temperature, the impact of *D* on xylem cavitation was calculated using a modification of the model presented by Kavanagh *et al*. (2010). Xylem water potential (Ψ_x_, in MPa) was calculated as shown in Eqn 3 earlier. PLC was then calculated as:

(Eqn 5) $$\text{PLC}=100/\left(1+\text{exp}(a\left(\Psi_{x}-b\right))\right)$$

where *a* and *b* are constants derived from a sigmoidal fit from the measured vulnerability curves (Pammenter & van der Willigen, 1998), with *b* representing the xylem water potential at 50% loss of conductance, or *P*_50_. For this simulation, Ψ_s_ was assumed to be −0.01 MPa, as cut shoots had been placed in water before the plume treatments.

As the increase in *D* was a consequence of increasing *T*, we adjusted PLC for the effects of a decrease in the surface tension of water, which has been shown to increase vulnerability to cavitation (Michaletz *et al*., 2012). We calculated the proportional drop in surface tension with temperature, and applied this correction to our PLC calculations:

(Eqn 6) $${\text{PLC}}_{\mathrm{st}}=\text{PLC}/\left(\sigma /\sigma_{20}\right)$$

where PLC_st_ is the PLC corrected for surface tension effects, σ is the surface tension of water (Vargaftik *et al*., 1983) and σ_20_ is the surface tension of water at 20°C.

We modelled two scenarios to provide an upper and lower bound for the sigmoidal PLC response to temperature. These bounds represent a range of possible sigmoidal solutions, rather than a general uncertainty bound in which any possible arrangement of data is supported by the model. For the upper bound, we assumed that stomatal conductance remained at its starting value for the duration of the plume exposure, representing the maximum potential water loss during the plume. For the lower bound, we assumed that stomatal conductance declined to zero following the initial model iteration, leaving only cuticular conductance (*g*_c_) as the pathway for leaf water loss for subsequent iterations. For each scenario, the model was iterated, reducing *K*_leaf_ by the calculated PLC at each iteration, until a stable result was obtained. Thus, the calculated PLC at each temperature represented exposure to only that temperature, and did not include cumulative effects of exposure to prior temperatures.

Another pathway of reduction in *K*_leaf_ during fire is via heat‐induced deformation of conduits (Michaletz *et al*., 2012). However, this was not included in our model as it has not been shown to exert a strong influence on the vulnerability curve and thus PLC (Michaletz *et al*., 2012). Thus, although heat‐induced deformation can contribute to permanently reduced *K*_leaf_ after the heating treatment (Michaletz *et al*., 2012), we viewed this as mainly influencing post‐plume recovery, rather than cavitation during the plume.

### Statistics

Significance between *K*_leaf_ treatments was determined using ANOVA followed by Tukey's honestly significant difference (HSD) *post‐hoc* analysis. Analyses were performed in JMP 8.0.2 (SAS Institute Inc, Cary, NC, USA).

## Results

### Experiment

**Figure 1:**
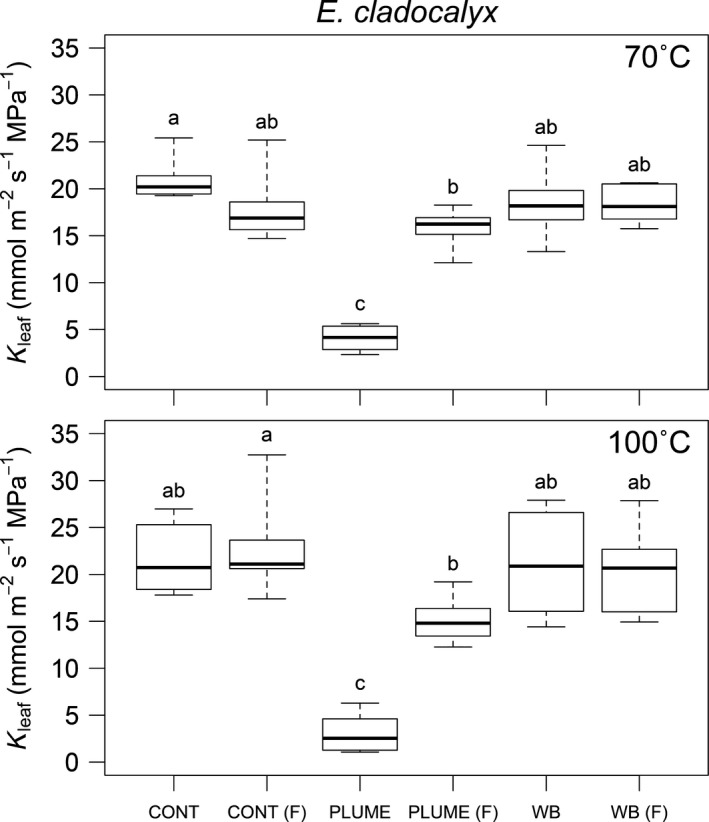
Leaf specific canopy conductance (*K*_leaf_, ± 1SE) measured for *Eucalyptus cladocalyx* shoots following the control (CONT), simulated heat plume (PLUME) and water bath (WB) treatments at 70 and 100°C. Following initial measurement, stems were flushed (F) and re‐measured to determine whether any loss of *K*_leaf_ was recoverable. Means were tested by ANOVA (Table 1). Significantly different means (*post‐hoc* Tukey's honestly significant difference (HSD) test) are indicated with unique letters.

**Figure 2:**
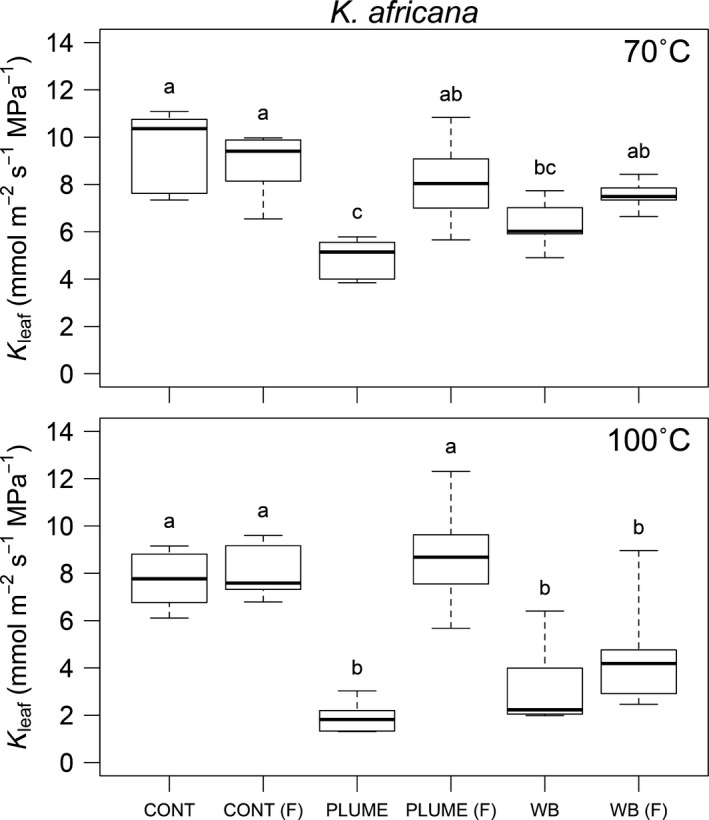
Leaf specific canopy conductance (*K*_leaf_, ± 1SE) measured for *Kiggelaria africana* shoots following the control (CONT), simulated heat plume (PLUME) and water bath (WB) treatments at 70 and 100°C. Following initial measurement, stems were flushed (F) and re‐measured to determine whether any loss of *K*_leaf_ was recoverable. Means were tested by ANOVA (Table 1). Significantly different means (*post‐hoc* Tukey's honestly significant difference (HSD) test) are indicated with unique letters.

There was no difference in *K*_leaf_ between the initial and flushed control stems for either *E. cladocalyx* or *K. africana* (Figs 1, 2; Table 1), indicating that our flushing procedure did not result in an over‐ or underestimation of *K*_leaf_.

**Table 1:** Results of ANOVA tests on leaf specific canopy conductance (*K*_leaf_) data

	*Eucalyptus cladocalyx*		*Kiggelaria africana*	
	70°C	100°C	70°C	100°C
*F*	29.6	18.5	10.1	17.6
*P*	< 0.0001	< 0.0001	< 0.0001	< 0.0001
df	35	34	33	35

**Figure 3:**
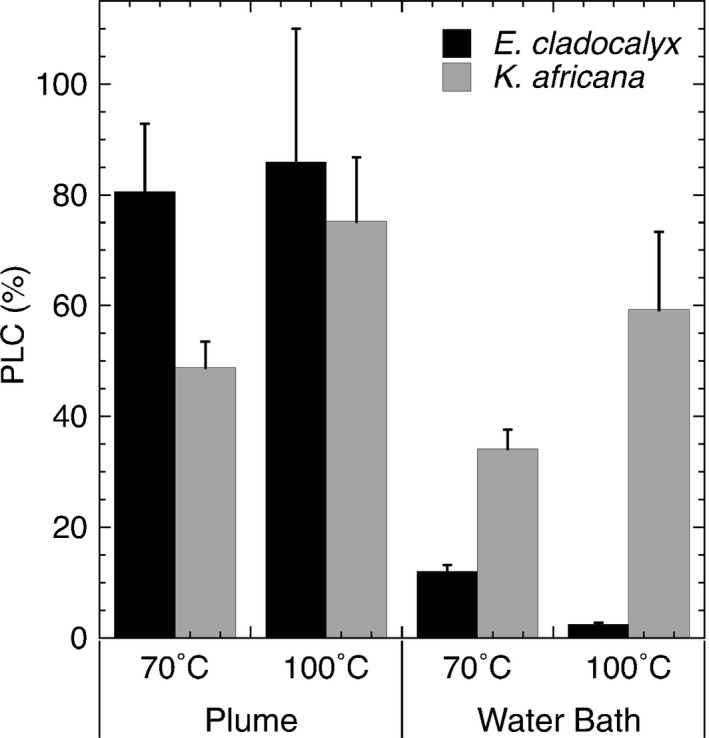
Percentage loss of conductance (PLC), calculated as the leaf specific canopy conductance (*K*_leaf_) of the unflushed treatment relative to *K*_leaf_ of the unflushed control (± 1SE), in *Eucalyptus cladocalyx* and *Kiggelaria africana*.

The simulated plume treatment resulted in significant loss of conductance relative to the control stems for both species at both 70 and 100°C (Figs 1, 2; Table 1). Approximately 80% loss of conductance was seen in both species when subjected to a 6‐min 100°C heat plume (Fig. 3). However, *K. africana* had significantly lower PLC (49%) in the 70°C treatment than *E. cladocalyx* (80%, Fig. 3). For all plume treatments, the loss of conductance was recoverable on flushing, as plume flushed stems were not significantly different from control flushed stems (Figs 1, 2; Table 1). The only exception was the *E. cladocalyx* 100°C plume treatment, where the plume flushed *K*_leaf_ was significantly different from the control flushed *K*_leaf_ (Fig. 1). However, it was not significantly different from the unflushed control, or from the flushed water bath treatment, indicating that this was, at best, a small difference.

There was no impact of the water bath treatment on *E. cladocalyx*. *Eucalyptus cladocalyx K*_leaf_ did not differ from the control in either the 70 or 100°C treatment, or in the post‐treatment flushed stems (Fig. 1). By contrast, the water bath treatment caused significant reductions in *K*_leaf_ for *K. africana*, which were comparable in magnitude to those seen in the plume treatments (Fig. 2). There was a greater loss of *K*_leaf_ in the 100°C water bath treatment than in the 70°C treatment (Figs 2, 3). The flushed 70°C stems were not different from the controls, indicating a full recovery of *K*_leaf_ (Fig. 2). However, the loss of *K*_leaf_ was not recoverable in the flushed 100°C treatment (Fig. 2), indicating possible permanent damage from heat‐induced deformation of the xylem in *K. africana*.

**Figure 4:**
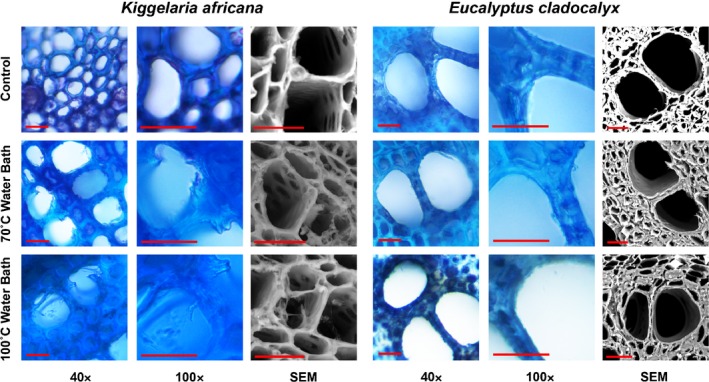
Light (× 40 and × 100 magnification) and scanning electron microscopy (SEM) of stem cross‐sections, showing xylem damage to *Kiggelaria africana* in 70 and 100°C water bath treatments, but not to *Eucalyptus cladocalyx*. Bars, 20 μm.

Physical damage to the xylem was examined using microscopy. Both light microscopy and SEM showed no damage to the xylem in *E. cladocalyx* for any of the treatments (Fig. 4). There was also no evidence of damage for the plume treatments in *K. africana* (Fig. 4). There was, however, indication of physical damage to the xylem of *K. africana* for both water bath treatments, with considerably more damage being visible in the 100°C treatment (Fig. 4).

**Figure 5:**
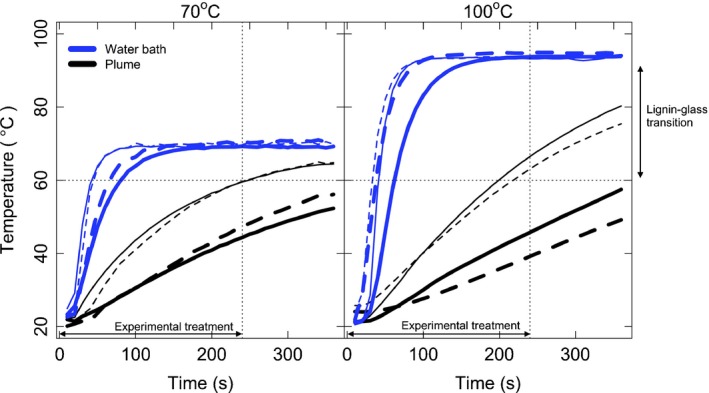
Temperature–time curves for *Kiggelaria africana* (solid lines) and *Eucalyptus cladocalyx* (dashed lines) for thin (4–5 mm, thin lines) and thick (8–9 mm, thick lines) stems in the 70 and 100°C simulated plume (black lines) and water bath (blue lines) treatments. Note the water bath treatments heat up far more rapidly than the plume treatments, and remain above 60°C for longer than the plume treatments. In the plume treatments, only the thin stems heated to above 60°C (the minimum temperature for the lignin glass transition) during the 6‐min experimental treatment.

The embedded thermocouples in the stems showed that only the thinner stems reached temperatures sufficiently high to attain the lignin glass transition temperature (60–90°C) in the plume treatments (Fig. 5). As expected, the 100°C plume treatment resulted in stems that were hotter, for longer, but the thicker stems still did not warm to over 60°C during the 6‐min plume simulation (Fig. 5). Stems in the water bath treatments warmed considerably faster, resulting in both thick and thin stems reaching the target temperature within the duration of the 6‐min exposure (Fig. 5).

### Model

**Figure 6:**
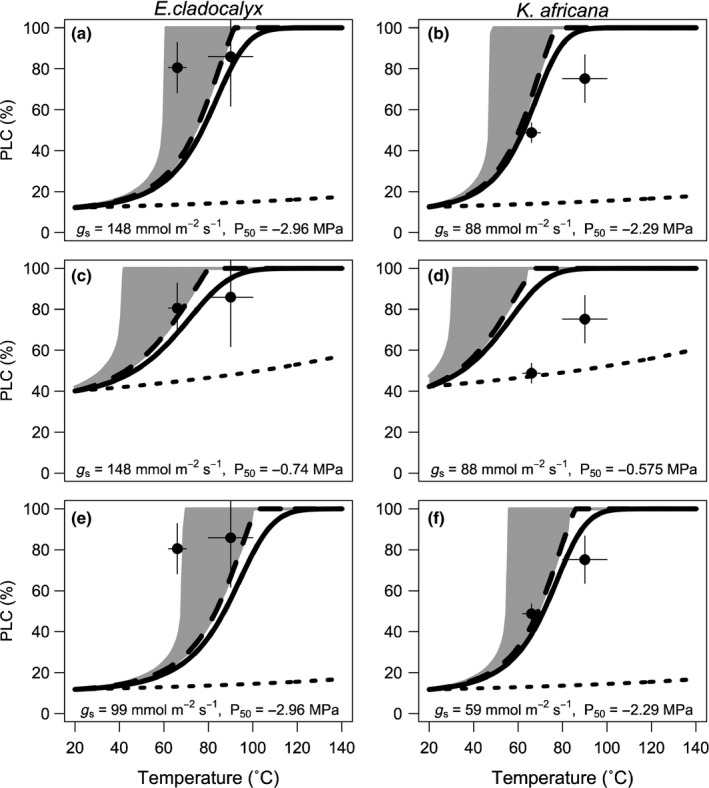
Model simulations of the percentage loss of conductance (PLC) vs temperature compared with experimental data (solid symbols) from the simulated plume treatments in *Eucalyptus cladocalyx* and *Kiggelaria africana*. For a full description of the model, see the Materials and Methods section and model diagram (Supporting Information Fig. S1). For each panel, the solid black line is the initial model result with no surface tension decrease (Fig. S1, box iii), the dashed black line includes surface tension effects (Fig. S1, box v), the shaded area represents the range of possible solutions encompassing assumptions of constant canopy stomatal conductance (*g*_s_) (Fig. S1, Loop 1) and rapid decline of *g*_s_ to cuticular conductance (*g*_c_) (Fig. S1, Loop 2), and the dotted black line is the effect of decreased surface tension only (holding *D* constant). (a, b) Model results with no parameter adjustments. (c, d) Model results after reducing *P*_50_ to simulate PLC in petioles. (e, f) Model results after reducing initial *g*_s_.

Our model simulation showed that the most significant cause of post‐fire loss of conductance was exposure to a high‐*D* plume, rather than a decrease in the surface tension of water in the xylem (Fig. 6). Assuming a constant *D*, and allowing the surface tension of water to decrease with temperature, very little change in PLC was found (Fig. 6, dotted line). Assuming a constant RH, and allowing *D* to track temperature, a rapid increase in PLC was observed (Fig. 6, solid black line), which was enhanced by including the effects of reduced surface tension of xylem water at equilibrium from that at air temperature (Fig. 6, dashed black line). In our experiment, the xylem temperature did not reach equilibrium with air temperature during the heat plume treatments (Fig. 5). As such, our full model (Fig. 6, dashed black line) may overestimate the surface tension effect on PLC. The actual PLC may fall closer to the *D*‐only model (Fig. 6, solid black line), where only air temperature, and not xylem temperature, is a factor. The small range between these models may be regarded as an uncertainty bound, influenced by the degree to which the xylem reaches equilibrium with air temperature.

The modelled PLC was strongly influenced by stomatal behaviour. Assuming a constant *g*_s_ throughout the plume, and allowing *K*_leaf_ to be reduced in concert with PLC, resulted in predictions of a rapid loss of conductance at relatively low temperatures (Fig. 6, upper bound of grey envelope). This represents an upper bound of possible PLC. However, it is unlikely that *g*_s_ would remain at a maximum throughout the plume as stomatal guard cells would desiccate, reducing *g*_s_. We thus also modelled a lower bound of possible PLC by rapidly reducing *g*_s_ to *g*_c_ (Fig. 6, lower bound of grey envelope).

Using the measured values of *K*_leaf_, *g*_s_ and vulnerability to cavitation, our model simulations appeared to underestimate PLC for *E. cladocalyx* and to overestimate PLC for *K. africana* (Fig. 6a,b). For *E. cladocalyx*, the model underestimated PLC at 70°C, except under the constant *g*_s_ scenario. Under this scenario, PLC at 100°C appeared to be overestimated, because of the inherent sigmoidal shape of the model. However, considering that PLC at 100°C for *E. cladocalyx* was not significantly different from 100% (*P* = 0.29), a sigmoidal curve saturating after 70°C would fall through the experimental uncertainty. Nevertheless, a more convincing solution is found when simulating hydraulic segmentation (i.e. localizing hydraulic failure to the distal shoots and petioles) by reducing the assumed *P*_50_. Distal shoots and petioles are known to be up to 20–60% more vulnerable to cavitation than are subtending stems (Johnson *et al*., 2009; Bucci *et al*., 2012, 2013; Charra‐Vaskou *et al*., 2012). For our model, a value of 25% of the measured *P*_50_ provided a reasonable fit for *E. cladocalyx* (Fig. 6c).

For *K. africana*, the initial model underestimated PLC at 100°C (Fig. 6b). This was not improved by simulating hydraulic segmentation (Fig. 6d). Instead, assuming an initial *g*_s_ of two‐thirds of the measured *g*_s_, we obtained a lower predicted PLC (Fig. 6e,f). Reducing the initial *g*_s_ considerably improved the model fit for *K. africana* (Fig. 6f), but not for *E. cladocalyx* (Fig. 6e).

## Discussion

Our study provides experimental and theoretical evidence for hydraulic failure during fire‐induced heat plumes and contributes to the growing evidence of a hydraulic mechanism for post‐fire mortality in trees (Balfour & Midgley, 2006; Kavanagh *et al*., 2010; Midgley *et al*., 2011; Michaletz *et al*., 2012). Both of the species examined in our study suffered from *c*. 80% loss of conductance when exposed to a simulated heat plume of 100°C. PLC of this magnitude is often fatal for angiosperms (Urli *et al*., 2013). This loss of conductance was fully recoverable on flushing, indicating that it was caused by air emboli, rather than permanent deformation of the xylem. Some degree of cavitation when exposed to a high‐*D* plume may be a biophysical inevitability (Fig. S1). If one assumes that stomata are unable to close sufficiently rapidly (Brodribb *et al*., 2009) to prevent a burst of transpiration in a fast‐moving heat plume, exposure to a plume of high *D* is likely to cause extensive cavitation. Importantly, even when assuming a rapid reduction of *g*_s_ to *g*_c_, extensive cavitation was predicted (Fig. 6). Furthermore, cavitation in the stem was not highly sensitive to direct heating of the stem, as shown by our model assuming constant stem temperature and thus surface tension (Fig. 6, solid line). Thus, importantly, cavitation should still occur for species well insulated by bark that might otherwise avoid lethally high stem temperatures, resulting in either cambium necrosis or xylem deformation. Post‐fire survival may thus depend on the location and extent of the cavitation and whether hydraulic function can be regained, possibly through epicormic resprouting (Clarke *et al*., 2013) or refilling of cavitated xylem (Hacke & Sperry, 2003; Salleo *et al*., 2004; Nardini *et al*., 2008; Martorell *et al*., 2014). Plants that are able to protect most of their hydraulic continuum during a fire plume should stand a better chance of being able to recover post‐fire. Later, we discuss possible mechanisms for such protection.

Despite having a more negative *P*_50_ in distal stems, *E. cladocalyx* experienced greater PLC than *K. africana* at 70°C (Figs 1, 2), which was contrary to our expectations. There was also no difference in PLC at 70 and 100°C for *E. cladocalyx*, indicating a highly vulnerable response to the simulated heat plume (Fig. 6a). Model simulations (Fig. 6) suggest that these results might be most parsimoniously explained by differences in hydraulic segmentation between the species.

Distal shoots and petioles have been shown to be more vulnerable to cavitation than are subtending stems (Johnson *et al*., 2009; Bucci *et al*., 2012, 2013; Charra‐Vaskou *et al*., 2012). Our *K*_leaf_ measurements were made on shoots that included more distal portions of the hydraulic pathway (including petioles) than those measured for *P*_50_. Thus, it is plausible that our measured *P*_50_ could overestimate the *P*_50_ for the entire shoot. When parameterized for an approximate petiole vulnerability curve (assuming *P*_50_ to be 25% of that of the subtending stem), our model was better able to explain the response of *E. cladocalyx* (Fig. 6c). This was not the case for *K. africana*, which was better predicted without the simulated petiole *P*_50_ (Fig. 6f). As a result, we speculate that the high PLC seen in *E. cladocalyx* was localized in the more vulnerable petioles, thereby protecting the main stem from cavitation.

The cavitation of distal shoots or petioles has been described as a ‘hydraulic fuse’ (Bucci *et al*., 2012; Nardini & Luglio, 2014), protecting the upstream xylem from cavitation. If the majority of the hydraulic continuum has been protected through such effective hydraulic segmentation (Zimmerman, 1983), the resprouting of new distal shoots or the refilling of embolized distal xylem may restore full hydraulic function. For *E. cladocalyx*, it is possible that both mechanisms may operate to enable post‐fire survival. It has been well established that *E. cladocalyx* is able to resprout epicormically (Burrows, 2000). There is also recent evidence of refilling after extreme drought in *Eucalytpus pauciflora* (Martorell *et al*., 2014), which might apply to other *Eucalyptus* species.

The lack of evidence for hydraulic segmentation in *K. africana* suggests that *K. africana* may experience a greater degree of cavitation in more proximal stems than does *E. cladocalyx*. Although a lack of segmentation may convey benefits of reduced PLC under natural dry atmospheric conditions, or even under a mild plume (as seen at 70°C), under conditions of extreme *D*, it would leave the stem susceptible to a greater degree of cavitation in the more proximal regions, leading to greater potential canopy die‐back.

An alternative explanation for the difference in PLC between the species at 70°C may be differences in capacitance, a factor not directly explored in this study. Theoretically, species with high capacitance may be able to avoid a rapid drop in water potential, and thus avoid extensive cavitation, during a short‐lived heat plume. However, the qualitatively similar wood and leaf properties of the species in this study suggest that differences in capacitance are unlikely to explain our observations. Nevertheless, we suggest that the investigation of the role of capacitance in protecting shoots from cavitation during a heat plume is an interesting avenue for future research.

There was clear evidence of permanent heat‐induced xylem deformation for *K. africana*, but not for *E. cladocalyx*. *Kiggelaria africana* showed reduced *K*_leaf_, which was not recoverable on flushing (Fig. 2), as well as visible signs of damage to the xylem (Fig. 4) after being heated in the water bath treatments. This fits the proposed mechanism of thermal softening of lignin that results in conduit deformation, or occlusion of pits, that is permanently solidified on cooling (Michaletz *et al*., 2012). Although there was no such sign of heat impact for *E. cladocalyx*, is it important to note that the water bath treatments were not conducted with stems under negative tension. Thus, the magnitude of possible deformation may be underestimated for actively transpiring trees (Michaletz *et al*., 2012). However, in our simulated heat plumes, extensive cavitation occurred before the stems reached the lignin glass transition temperature (Fig. 5). In addition, extensive leaf scorch may have halted transpiration. In this case, the stems may not be experiencing increasing levels of tension by the time that they reach the thermal softening point. Currently, little is known about the interaction of cavitation and thermally induced conduit collapse (Michaletz *et al*., 2012). However, regardless of the absolute magnitude of deformation seen in our experiment, our results clearly indicate that *E. cladocalyx* was considerably less susceptible than *K. africana* to the direct effects of heating. Sensitivity to heat of the xylem may be a crucial trait for post‐fire recovery. Species that experience a thermal softening of lignin at higher temperatures, or have an anatomy that prevents lignin from occluding pit pores, or altering cell geometry, may be more resistant to permanent hydraulic failure post‐fire.

Lastly, our model showed that the response to *D* was very sensitive to initial *g*_s_. Species that encounter the fire plume with a lower *g*_s_, or rapidly reduce *g*_s_ on exposure to the plume, were predicted to experience lower PLC (Fig. 6e,f). It will be interesting to see to what extent this model prediction is supported by observation, but it raises the possibility that leaf traits may be important in determining the cavitation response in a fire plume. The impact of desiccation on *g*_s_ and *K*_leaf_ varies considerably between species (Brodribb & Holbrook, 2006; Sack & Holbrook, 2006; Brodribb *et al*., 2009), and is caused in part by the cavitation of leaf veins (Kikuta *et al*., 1997; Salleo *et al*., 2000; Nardini *et al*., 2001, 2003; Cochard *et al*., 2002; Brodribb & Holbrook, 2003; Johnson *et al*., 2011), as well as differences in vein architecture (Scoffoni *et al*., 2011). There is also the intriguing possibility that smoke may provide a cue for stomatal closure that might mitigate the impact of a fire plume (Gilbert & Ripley, 2003; Calder *et al*., 2010; Bell *et al*., 2013; Aerts, 2015). However, our simulations were based on an assumption of a constant cuticular conductance that was unaffected by the heating of the leaf. If heating considerably increases *g*_c_, this would limit the role of stomatal control in influencing PLC during a heat plume.

### Conclusion: a suite of ‘pyrohydraulic’ traits?

Fire survival in trees is a complex process involving a suite of traits, including plant architecture, bark thickness and bud protection, amongst others (Keeley *et al*., 2011; Clarke *et al*., 2013). Our study contributes to the evidence for an important role of hydraulic traits in determining fire mortality or survival. We propose that three hydraulic traits may be particularly important. First, hydraulic segmentation may protect the main stem from cavitation, potentially allowing rapid recovery post‐fire. Second, the thermal sensitivity of the xylem will determine the extent to which the loss of hydraulic conductance following a heat plume can be recovered. Finally, the combination of stomatal and cuticular sensitivity to the heat plume may determine the extent of cavitation that the tree experiences. This suite of ‘pyrohydraulic’ traits, and possibly others, such as the role of capacitance in buffering water potential during short‐duration heat plumes, might help to explain why some tree species retain their canopy after low‐intensity fires, whereas others suffer extensive die‐back or are killed.

## Author contributions

A.G.W., W.J.B., J.J.M. and J.A.N. designed the research. J.A.N. collected the data. A.G.W. and J.A.N. performed data analysis and modelling. A.G.W., J.A.N., J.J.M. and W.J.B. interpreted the data. A.G.W. wrote the manuscript.

## Supporting information

Please note: Wiley Blackwell are not responsible for the content or functionality of any supporting information supplied by the authors. Any queries (other than missing material) should be directed to the *New Phytologist* Central Office.

**Fig. S1** Model diagram showing the link between plume exposure and percentage loss of conductance and leaf specific canopy conductance.
**Table S1** Values for initial model parameterization
