# Supplementary material for: Experimental evidence for heat plume‐induced cavitation and xylem deformation as a mechanism of rapid post‐fire tree mortality
Source: New Phytol. 2016 May 6;211(3):828–38. doi: 10.1111/nph.13979 (PMC5084795; doi:10.1111/nph.13979)
Supplement: Supplementary file 1 — Fig. S1 Model diagram showing the link between plume exposure and percentage loss of conductance and leaf specific canopy conductance. Table S1 Values for initial model parameterization [file NPH-211-828-s001.pdf]

New Phytologist

***New Phytologist* Supporting Information Fig. S1 and Table S1**

Article title: Experimental evidence for heat plume-induced cavitation and xylem deformation as a mechanism of rapid post-fire tree mortality

Authors: Adam G. West, Jacques A. Nel, William J. Bond and Jeremy J. Midgley

Article acceptance date: 18 March 2016

The following Supporting Information is available for this article:

Fig. S1 Model schematic showing the link between plume exposure and percent loss of conductance and leaf specific canopy conductance.

Table S1 Values for initial model parameterization

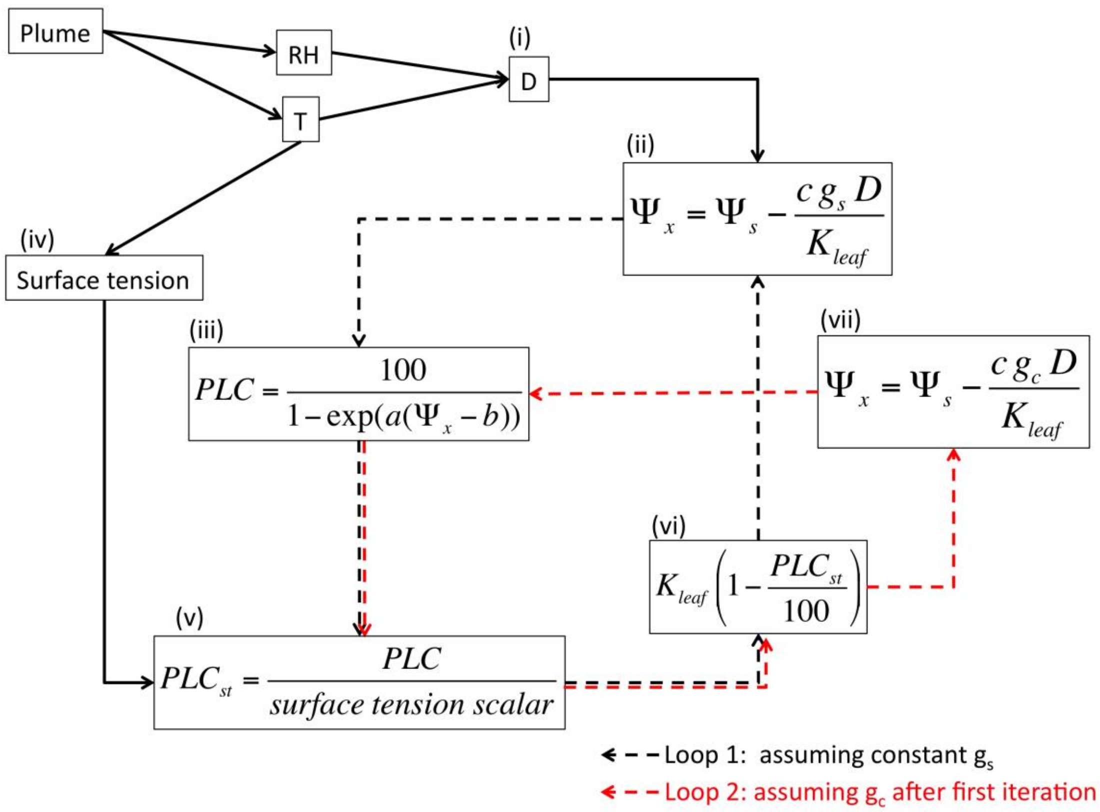

Fig. S1 Model schematic showing the link between plume exposure and percent loss of conductance (PLC) and leaf specific canopy conductance (K_{leaf}). The heat plume results in low relative humidity and high temperature, which combine to influence vapour pressure deficit (D, box i). D influences xylem pressure (Ψ_x, box ii), resulting in a loss of conductance (PLC, box iii), which is further exacerbated by reductions in surface tension with temperature (boxes iv, v), resulting in a reduced K_{leaf} (box vi). The model can then be looped to simulate continued transpiration assuming constant canopy stomatal conductance (g_s) (box ii, Loop 1) or allowing g_s to immediately drop to a minimum of cuticular conductance (g_c, box vii, Loop 2), until a stable result is achieved.

New Phytologist

Table S1 Values for initial model parameterization

Parameter (units)	*E. cladocalyx*	*K. africana*
a	0.708	0.951
b (MPa)	-2.96	-2.29
g_s (mmol m^{-2} s^{-1})	148	88
Ψ_s (MPa)	-0.01	-0.01
K_{leaf} (mmol m^{-2} s^{-1} MPa^{-1})	21	8.5
g_c (mmol m^{-2} s^{-1}) *	5	5

*__Nobe PS. 2009.__ *Physiochemical and environmental plant physiology. 4th edn.* Amsterdam, the Netherlands: Elsevier.
